# Meat quality, post-mortem proteolytic enzymes, and myosin heavy chain isoforms of different Thai native cattle muscles

**DOI:** 10.5713/ab.20.0798

**Published:** 2021-04-23

**Authors:** Chanporn Chaosap, Panneepa Sivapirunthep, Ronachai Sitthigripong, Piyada Tavitchasri, Sabaiporn Maduae, Tipyaporn Kusee, Jutarat Setakul, Kazeem Adeyemi

**Affiliations:** 1 Department of Agricultural Education, Faculty of Industrial Education and Technology, King Mongkut’s Institute of Technology Ladkrabang, Bangkok 10520, Thailand; 2 Department of Animal Technology and Fishery, Faculty of Agricultural Technology, King Mongkut’s Institute of Technology Ladkrabang, Bangkok 10520, Thailand; 3 Department of Animal Science, King Mongkut’s Institute of Technology Ladkrabang, Prince of Chumphon Campus, Chumphon 86160, Thailand; 4 Department of Animal Production, Faculty of Agriculture, University of Ilorin, PMB 1515, Ilorin, Nigeria

**Keywords:** Calpain, Calpastatin, Collagen, Glycogen, Troponin

## Abstract

**Objective:**

This study investigated the meat quality characteristics, endogenous proteolytic enzymes, collagen content, and myosin heavy chain (MyHC) isoforms of different muscles of Thai native cattle (TNC).

**Methods:**

*Infraspinatus* (IF), *Longissimus thoracis* (LT), and *Supraspinatus* (SS) muscles were obtained from two TNC breeds, Kho-Lan (KL, n = 7) and Kho-Isaan (KI, n = 7). The muscle and meat characteristics of TNC breeds and their relationship with MyHC expression were examined.

**Results:**

Three MyHC isoforms namely MyHC I, MyHC IIa, and MyHC IIx were detected in the muscles. The KL had higher (p<0.05) MyHC IIx than the KI. The IF muscle had higher (p<0.05) MyHC I compared to other muscles. The LT muscle had the least MyHC I. The LT had higher (p<0.05) MyHC IIx than the IF and SS muscles. The IF presented the least MyHC IIx. The KL had higher (p<0.05) lightness and moisture content and lower crude protein, redness, cooking loss, shear force, and calpastatin than the KI. The glycogen, total collagen, soluble collagen, crude protein, ash contents, and troponin T degradation product of IF and SS were lower (p<0.05) than that of LT. Ether extract in LT was lower (p<0.05) than that of IF and SS. The percentage of MyHC I, MyHC IIa, and MyHC IIx were significantly correlated with muscle and meat characteristics of TNC.

**Conclusion:**

These results suggest that the differences in the MyHC isoforms may partly account for the variation in meat quality between breeds and among muscles of TNC.

## INTRODUCTION

The beef cattle population in Thailand recently is about 5.40 million heads. Beef cattle in Thailand can be classified into three groups according to their genetic types, Thai native cattle (TNC)/crossbreds 56.60%, purebreds/crossbreds 40.47%, and fattened cattle 2.93% [1]. The TNC are one of the most economically important domestic animals in Thailand raised by smallholder farmers in mixed crop-livestock systems [1,2]. The TNC have characteristic of *Bos indicus* (Zebu), including the distinctive dorsal hump [1]. Despite being small and having a slow growth rate, TNC are well adapted to harsh and humid climates. Hence, they have high resistance to ticks, other parasites, and diseases [3]. They are capable of utilizing agricultural by-products or low quality ingredients such as feeds as well as having high fertility and good mothering ability [3]. There are four native breeds officially recognized by the Department of Livestock Development, Ministry of Agriculture and Cooperatives, Thailand, namely Kho-Khaolumpoon (Northern Thailand), Kho-Isaan (KI, Northeastern Thailand), Kho-Lan (KL, Central Thailand), and Kho-Chon (Southern Thailand) [2]. They are distinct from each other by phenotypic and genetic traits as well as geographical regions of origin [2].

Thai native beef has less fat, greater proportion of polyunsaturated fatty acids, high conjugated linoleic acids and low cholesterol compared to *Bos taurus* crossbreds thereby making it a favorable option for health-conscious consumers [4]. In addition, it is widely known among local meat processors that Thai native beef offers excellent functionality due to its high water retention capacity in processed traditional Thai meat products such as Look Chin or meatball and Nham or fermented meat. However, Thai native beef is tougher compared to crossbred of *Bos taurus* [4]. In spite of the established genetic and phenotypic differences among the TNC, the breed-induced variations in meat quality of the TNC remain unclear. In addition, information on muscle fiber types and metabolic properties of different muscles, which underpin the meat quality of TNC is very rare. Elucidating the muscle fiber type of different muscles would yield useful information as to the cuts of TNC meat that could be best suited for particular processing conditions. The functional and biochemical characteristics of each muscle would likely necessitate different processing conditions for optimum utilization of muscles in value-added products [5].

Skeletal muscles consist of different muscle fiber types, varying in biochemical and structural characteristics, which can influence meat quality attributes [6]. Muscle fiber types and the different isoforms of the myosin heavy chain (MyHC) are reliable markers of muscle fiber diversity [7,8]. Calpain systems are the major proteolytic enzymes affecting meat tenderness [9]. Currently, there is limited data on the meat quality characteristics, postmortem proteolytic enzymes, and muscle fiber classification of TNC. The TNC used in this study were KI and KL from northeastern region and central region, respectively. The northeastern region and central region are respectively the first and second largest region for beef cattle production in Thailand [1]. The objectives of this study were to access the meat quality characteristics, endogenous proteolytic enzymes, collagen content, and MyHC isoforms as indicators of muscle fiber type as well as metabolic properties of different muscles of TNC.

## MATERIALS AND METHODS

### Animal ethics

Muscle samples used in this study were obtained from two sources, KL cattle from central region Karnjanaburi province which was part of Government projects, sponsored by the Thailand Research Fund (project code RDG5220024) and KI cattle from northeastern region Ubonratchatani province (project code RDG5220025). All experimental procedures were carried out following the animal welfare standards of the Animal Care and Use Committee of Department of Livestock Development, Ministry of Agriculture and Cooperatives, Royal Thai Government.

### Experimental cattle, slaughtering procedure, and muscle sample collection

The TNC, KL, and KI, seven entire male cattle per each breed were used in this study. The cattle were extensively grazed on natural forage until they reached the target slaughter weight at approximately 150 kg and the average age was approximately 2 years. All cattle were kept in lairage with access to water for 12 hours prior to slaughter. Weighting, stunning, bleeding, skinning, evisceration, and washing were completed while the carcasses were hung on achilles on an overhead rail. Dressed carcasses were then weighed before being split longitudinally. Muscle samples from *Infraspinatus* (IF), *Longissimus thoracis* (LT), and *Supraspinatus* (SS) muscles were removed from the left side of each carcass within approximately 1 h postmortem. The samples were trimmed free of all visible fat, cut into 50 g pieces, snap frozen in liquid nitrogen and kept at −80°C until subsequent calpain systems, glycogen content, and MyHC expression analysis. Simultaneously, the same muscle samples were removed from the right side of each carcass at approximate 1 kg weight and transported in the icebox to meat science and technology laboratory, Faculty of Agricultural Technology, King Mongkut’s Institute of Technology Ladkrabang, Thailand and kept in a walk-in chiller (2°C±2°C) for 48 h postmortem before measuring color and pH. Each muscle sample was cut longitudinally into two 3 cm thickness sub samples, the sub-samples were vacuum packaged, and stored at −20°C until subsequent shear force, nutrient composition, collagen content, and troponin T degradation analyses.

### Analysis of myosin heavy chain expression

Muscles of cattle known to express specific MyHC isoforms [8] were used as controls: *masseter* (MA) (MyHC I only), *cutaneus trunci* (CT) (MyHC IIa and IIx), *diaphragm* (DI) (MyHC I and IIa), and *trapezius* (TZ) (MyHC I, IIa, and IIx). These were obtained from TNC. For each muscle sample, MyHC isoforms were separated by gel electrophoresis following the method of Picard et al [10].

### Casein zymography

The activities of calpain 1 and calpain 2 were analyzed using casein zymography according to the method described by Arther and Mykles [11]. A 200 mg of the crushed sample was homogenized in 2 mL of extraction buffer (50 mM Tris/HCl [pH 7.5], 5 mM ethylenediaminetetraacetic acid (EDTA), 200 μg/mL of 2-[4-aminoethyl]-benezenesulphonyl fluoride, 1 μg/mL of leupeptin, 1 μg/mL of pepstatin). After centrifugation at 15,000×g for 20 min at 4°C, the supernatant was collected and mixed with 1:1 with sample buffer (125 mM Tris-HCl pH 6.8, 0.1 M 1,4-dithiothreitol (DTT), 20% [v/v] glycerol, 0.01% [w/v] bromophenol blue). The mixed supernatant was loaded onto a non denaturing gel consisting of 2% (w/v) casein incorporated into 10% (w/v) acrylamide separating gel with 5% (w/v) stacking gel without casein. The gels were pre-run for 30 min at 4°C in electrophoresis buffer (25 mM Tris, 125 mM glycine, 1 mM EDTA, 1 mM DTT, pH 8.3) at 125 V. Samples were loaded at approximately 100 μg of protein with equal wet weight of tissue equivalent per well and electrophoresed on gels under pre-run conditions at 125 V for 4 h at 4°C. Thereafter, gels were incubated in buffer containing 50 mM Tris-HCl, pH 7.0, 5 mM CaCl_2_ and 10 mM DTT with three changes. Then gels were fixed in 10% (v/v) acetic acid for 10 min, stained with Coomassie staining solution for 30 min and destained with 10% (v/v) acetic acid. The bands in the gel were quantified using Quantity One Multi Analyst imaging software (Bio-Rad, Hercules, CA, USA).

### Calpastatin by iElisa

Calpastatin was measured by iElisa using anti-calpastatin (in house), before using in house antibody for sample analysis. Prior to that, the in house anti-calpastatin was tested against calpastatin antigen (208902, Millipore Merck, Billerica, MA, USA), which is located in a region found in all calpastatins, and the result was compared with commercial antibody (clone 1F7E3D10, Sigma, St. Louis, MO, USA). The result showed a highly significant correlation between in house antibody and commercial antibody (r = 0.964, p<0.01).

A portion of the frozen muscle (approximately 10 g) was weighed and extracted in 2 volumes of ice-cold extraction buffer (100 mM Tris/HCl; pH 8.0; 10 mM EDTA). Tissue was homogenized for 15 s using a polytron on high speed. The homogenate was centrifuged at 16,000×g for 15 min at 4°C. After centrifugation, the supernatant fraction was collected, heated at 95°C for 5 min, cooled on ice for 10 min, and then centrifuged at 16,000×g for 15 min at 4°C. Protein concentrations of the heated supernatant fraction were determined by the method of Lowry et al [12]. The heated muscle homogenates were diluted to 4 mg/mL in phosphate buffer saline (PBS). The diluted samples (100 μL/well) were incubated in a 96 well plate (Maxisorb; Nunc, Roskilde, Denmark) for 2 h at 37°C. Wells were emptied and washed three times with PBS containing 0.05% Tween-20 (TPBS), blocked with 1% skim milk in TPBS for 1 h at 37°C and then incubated for 1 h at 37°C with 100 mL/well rabbit anticalpastatin antibody diluted to 30 mg/mL immunoglobulin G (IgG) in 1% BSA-TPBS. Wells were then washed three times with TPBS and goat anti-rabbit IgG-peroxidase conjugate (1:2,000 dilution; A-6154 Sigma-Aldrich, St. Louis, MO, USA) in 1% BSA-TPBS was applied for 1 h at 37°C. Following three washes with TPBS, 100 mL/well 3,3′,5,5′-tetramethylbenzidine (002023; Invitrogen, Carlsbad, CA, USA) substrate was applied, and this resulted in formation of a soluble blue product. After 15 min at 37°C, the reaction was stopped by adding 100 mL/well of 0.01% sodium dodecyl sulfate (SDS) to each well and absorbances were measured at 650 nm in a microplate reader (Infinite F50, Tecan, Männedorf, Switzerland).

### Western blot for anti-troponin T

Muscle tissue submerged in liquid nitrogen was crushed into powder and the western blot anti-troponin T was assessed as described by Chaosap et al [13]. A 300 mg of crushed sample was homogenized in 6 mL of extraction buffer (50 mM Tris/HCl pH 7.5, 5 mM EDTA, with protease inhibitors 200 μg/mL 2-(4-aminoethyl)-benzenesullphonyl fluoride, 1 μg/mL leupeptin, 1 μg/mL pepstatin for 60 s. Whole homogenate was added to an equal volume of 2× SDS sample mix (125 mM Tris-HCl pH 6.8, 4% [w/v] SDS, 0.1 M DTT, 20% [v/v] glycerol, 0.01% [w/v] bromophenol blue). Samples in SDS sample mix were boiled at 100°C for 5 min before loading onto pre cast 10% stain free gel with 5% stacking gel. Gels were run on a vertical dual plate unit at 200 mV in 1× SDS running buffer for approximately 45 min. The separated proteins on gels were transferred to PVDF membranes using a trans blot tank with electrodes (Bio-Rad, USA) filled with transfer buffer (400 mM glycine, 25 mM Tris, 5% [v/v] isopropanol) at a constant current of 200 mA for 2 h. Proteins transferred onto membranes were blocked with 5% milk (w/v) in TBS-T for 1 h at 25°C, subsequently probed by anti-troponin T diluted to 1:7,500 in 5% (w/v) milk TBS-T overnight at 4°C and washed with 1% (w/v) milk TBS-T for 6×5 min with fresh changes of solution. The membrane was then incubated for 1 h with anti-mouse conjugated with IgG horseradish peroxidase diluted to 1: 7,500 in 5% (w/v) milk TBS-T and washed with 1% (w/v) milk TBS-T as mentioned before. The proteins on membrane were detected with chromogenic substrates. The intensity of visually detectable bands was quantified using a Quantity one Multi Analyst imaging software (Bio-Rad, USA).

### Nutrient composition measurements

The meat samples were analyzed for dry matter (DM), crude protein (CP), ether extract (EE), and ash according to AOAC [14]. DM was determined at 105°C for 12 h followed by cold weighing. Nitrogen content was determined by the micro Kjeldahl method and was multiplied by 6.25 to determine CP. The EE was determined using petroleum ether in the Soxhlet apparatus. Ash was determined by placing sample in a furnace at 550°C for 6 h.

### Glycogen analysis

Muscle glycogen concentrations were determined as described by Dreiling et al [15]. Briefly, 200 mg of muscle sample was homogenized with 2 mL cold 8% (v/v) perchloric acid using a polytron (Ultra Turrax T25 disperser, IkA-Labortechnik, Staufen, Germany) and then centrifuged at 15,000×g for 10 min. The supernatant was neutralized with saturated sodium bicarbonate solution and 0.2 M sodium acetate buffer (pH 4.8). A glycogen standard curve was established by dissolving 0 to 90 μg of bovine liver glycogen (Cas no. 9005-79-2, Sigma-Aldrich, Darmstadt, Germany) in neutralized perchloric acid. Each standard and neutralized supernatant were incubated in amyloglucosidase solution (A7255, Sigma-Aldrich, Germany) for 30 min at 55°C to convert glycogen to glucose. The concentration of glucose was determined using Enzymatic Glucose Reagent (Infinity Glucose Oxidase Reagent, Thermo Scientific, Lidcomb, NSW, Australia), by measuring the absorbance at 550 nm on a 96-well microplate reader (Infinite F50, Tecan, Switzerland).

### Determination of collagen

The concentration of soluble, insoluble, and total collagen were determined as described by Hill [16]. Briefly, 0.9 g of the minced meat sample was homogenized with 5 mL of 1/4 strength Ringer’s solution. The homogenate incubated at 77°C for 60 min and then equilibrated at room temperature before centrifuging at 2,500×g for 10 min. The supernatant (soluble collagen) and pellet (insoluble collagen) were separated and hydrolyzed for 24 h at 110°C in 12 N HCL and 6 N HCl, respectively. The cool hydrolysate was added with activated carbon, mixed thoroughly, filtered through Whatman no.1 paper, adjusted pH to approximately 6.7, and diluted with distilled water to either 100 or 500 mL for soluble and insoluble assay, respectively. A hydroxyproline standard curve was prepared by dissolved hydroxyproline standard (56250, Sigma-Aldrich, Germany) in concentrations ranging from 1 to 7 mg/mL. Then, 400 μL of each standard or sample diluted hydrolysate was added 200 μL of freshly prepared Chloramine T hydrate (Cas no. 149358-73-6, Sigma-Aldrich, Germany) in an aqueous buffer solution (pH 6.0) which contained NaOH, citric acid, sodium acetate and 1-propanol and incubated at room temperature for 20 min. Then 200 μL of 4-dimethylamino-benzaldehyde reagent (4132845; Ehrlich’s reagent, Sigma-Aldrich, Germany) in an aqueous solution containing strong (70%) perchloric acid and 2-propanol was added and incubated at 60°C for 20 min then at 25°C for 20 min. Absorbance at 550 nm was measured with a microplate reader (Infinite F50, Tecan, Switzerland). Hydroxyproline content was determined using a standard curve for hydroxyproline with the collagen content being calculated from the hydroxyproline content using a conversion factor of 7.25.

### Meat quality measurements

The pH of the IF, LT, and SS muscles was measured at 48 h postmortem using a pH meter with a puncture electrode (Model SG2 - ELK Seven Go, Mettler-Toledo International Inc., Giessen, Germany).

The Cielab L* (lightness), a* (redness), and b* (yellowness) values of each muscle sample were measured using a chromameter (CR-300, Konica Minolta, Osaka, Japan) with an illuminant D65 light source. Three measurements were taken from each sample and the average was recorded.

For cooking loss, the samples were weighed, vacuum-sealed using plastic bags and then cooked in a constant 80°C water bath for 30 min or until core temperature of meat sample reached 70°C. The cooked samples were cooled down to room temperature before weighing. Cooking loss of meat sample was expressed as percent weight loss over the initial weight before cooking. Eight 1 cm×2 cm×1 cm slices were removed parallel to the fiber orientation through the thickest portion of the cooked muscle.

Warner-Bratzler shear force was determined by using a universal testing machine (model 2519-104, Instron, Norwood, Massachusetts, USA) equipped with a Warner-Bratzler shear head operating at a crosshead speed of 50 mm/min.

### Statistical analysis

Data were analyzed using the general linear model procedure of SAS (SAS Institute Inc., Cary, North Carolina, USA). The model included the effects of cattle breed (B), muscle (M), and B×M interaction and random residual error. Least-square means were separated by using the PDIFF option. The Pearson correlation coefficients were tested between MyHC expression and meat characteristics. The level of significant adopted was p<0.05.

## RESULTS

### Myosin heavy chain expression

**Figure 1:**
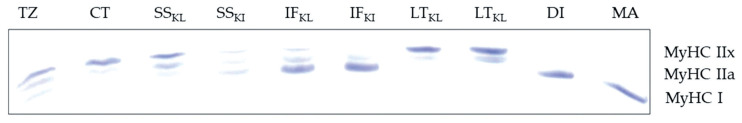
Electrophoresis profile of myosin heavy chain (MyHC) isoforms in *infraspinatus* (IF), *Longissimus thoracic* (LT), and *supraspinatus* (SS) muscles of Kho-Lan (KL) and Kho-Isaan (KI). Trapezius (TZ), cutaneous trunci (CT), diaphragm (DI), and masseter (MA) from Boer crossbred goat were used as controls to classify the isoforms.

**Figure 2:**
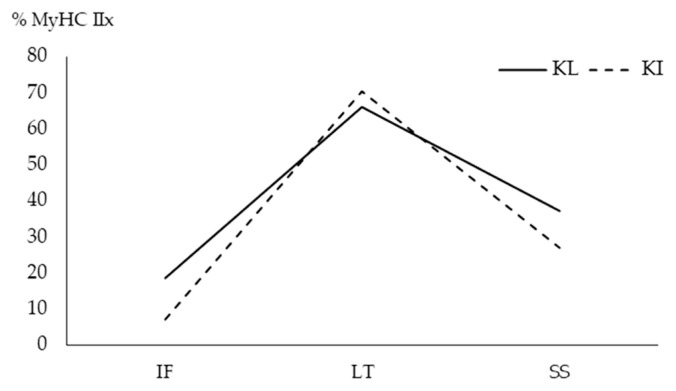
Interaction between Thai native cattle breeds and muscle types on relative percentage (%) of myosin heavy chain (MyHC) IIx present in *infraspinatus* (IF), *Longissimus thoracic* (LT), and *supraspinatus* (SS) muscles of Kho-Lan (KL) and Kho-Isaan (KI) determined by gel electrophoresis.

The MyHC isoforms in different muscles of KI and KL separated by gel electrophoresis are presented in Figure 1. Breed and muscle type interaction was not significant for the expression of MyHC I and MyHC IIa in TNC (Table 1). The expression of MyHC I was not influenced (p = 0.051) by TNC breed. The IF muscle had higher MyHC I (p<0.0001) compared with other muscles. The LT muscle had the least MyHC I. The expression of MyHC IIa was neither different between TNC breeds (p = 0.519) nor among muscle types (p = 0.285). There was a significant interaction (p = 0.038) between breed and muscle for MyHC IIx. As shown in Figure 2, MyHC IIx of KI was lower in IF and SS but higher in LT than that of KL.

### Calpain systems and degradation product of troponin T

There was no significant interaction between breed and muscle type on calpain activity, calpastatin concentration, and degradation product of troponin T in TNC (Table 2). Breed and muscle type did not affect calpain 1 and calpain 2 activity. Calpastatin content in KI was higher (p = 0.006) than that of KL. Calpastatin content did not differ among muscle types. Breed did not affect the relative percentage of the 30 kDa degradation product of troponin T on day 2 postmortem. The LT had higher (p = 0.004) troponin T degradation product than other muscles.

### Nutrient compositions and meat quality characteristics

There was no significant interaction between breed and muscle type for nutrient composition in TNC (Table 3). The KL had lower CP (p<0.0001) and higher moisture (p<0.0001) than KI. Breed had no effect on EE and ash in TNC beef.

As might be expected, there were significant differences between muscles for all the parameters assessed (p<0.05) with the slow type IF (high proportion of MyHC I expression) and fast type LT (high proportion of MyHC IIx expression) being at the extremes (Table 3). The CP of IF and SS was lower (p<0.0001) than that of LT. The EE of IF was lower (p = 0.0002) than that of LT. The EE of SS was similar to that of IF. The LT had lower moisture (p<0.0001) than IF and SS. Ash was higher (p<0.0001) in LT than in IF and SS.

**Figure 3:**
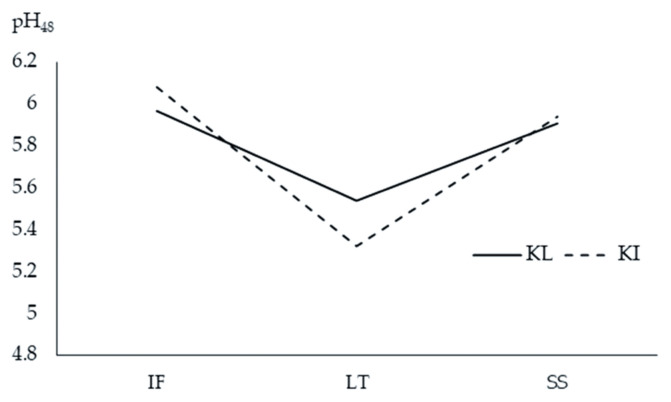
Interaction between Thai native cattle breeds and muscle types on pH_48_ of *infraspinatus* (IF), *Longissimus thoracic* (LT), and *supraspinatus* (SS) muscles of Kho-Lan (KL) and Kho-Isaan (KI).

There was no significant interaction between breed and muscle type on meat quality characteristics in TNC, except for pH_48_ (p = 0.023) as shown in Figure 3. The pH_48_ of IF and SS of both KI and KL did not differ (p>0.05). However, the LT of KI exhibited lower pH_48_ than did the LT of KL (p<0.05). Breed had no effect on muscle glycogen (p = 0.066), total collagen (p = 0.534), soluble collagen (p = 0.309), insoluble collagen (p = 0.429), and b* (p = 0.053) in TNC. The LT had higher glycogen (p = 0.037), lower insoluble collagen (p = 0.002), and total collagen (p = 0.002) and tended to have lower soluble collagen (p = 0.052) compared to other muscles. Total collagen and insoluble collagen did not differ between SS and IF. The KL had higher lightness (p<0.0001) and lower redness (p<0.0001), cooking loss (p = 0.013), and shear force (p<0.0001) than the KI. The IF had lower lightness (p<0.0001) and higher redness (p<0.0001) than did other muscles. Lightness did not differ between LT and SS. The LT had lower redness and yellowness (p<0.0001) compared with other muscles. Shear force was lower in IF and higher in LT (p< 0.0001) than other muscles.

### Relationships between myosin heavy chain isoforms and meat characteristics

The muscles selected in this study gave a range of MyHC expression particularly for MyHC I and MyHC IIx, relative to each other, IF was slow type muscle (high proportion of MyHC I expression) and LT being a fast type muscle (high proportion of MyHC IIx expression). Therefore, the relationship between MyHC isoform expression and the meat characteristics of the muscles was examined (Table 4). MyHC I had a positive correlation with insoluble collagen, total collagen, pH, a*, b*, % EE, and % moisture while it had a negative correlation with L*, shear force, % protein, and % ash (p<0.01). MyHC IIa negatively correlated with calpastatin (p<0.01). MyHC IIx negatively correlated with insoluble collagen, total collagen, pH, a*, b*, and % fat and positively correlated with L*, shear force, % protein, % ash, and the degradation product of troponin T (p<0.01).

## DISCUSSION

Skeletal muscles are composed of different types of muscle fibers, differing in their molecular, structural, contractile, and metabolic properties, which contribute to the differences in their functional capabilities and to the quality of meat [6,10]. The expression of MyHC gives an indication of the fiber type of the muscle with high proportions of MyHC I and MyHC IIx being associated with slow and fast type fibers, respectively [6]. The MyHC isoforms were identified with four control TNC muscles; cutaneous trunci (MyHC IIa and MyHC IIx), trapezius (MyHC I, MyHC IIa, and MyHC IIx), Diaphragm (MyHC I and MyHC IIa), and Masseter (MyHC I), whose MyHC isoform composition had been previously confirmed by comparing with ovine, camel, rat, and beef muscles [10].

The muscles of TNC examined in this study contained three MyHC isoforms (I, IIa, and IIx), which was similar to that observed in most large domestic adult mammals [8,17]. As expected, there was no detectable MyHC IIb in the muscles considered in the present study, as this isoform is not present in most large animals, except for certain specialized muscles in bovine [17,18]. As indicated by the relative percentage expression of MyHC I, the IF and to some extent the SS, could be considered to be slow type muscles. The LT muscle characterized by high MyHC IIx and low MyHC I could be considered a fast type muscle. The higher expression of MyHC IIx in the KL than in the KI may reflect the differences in growth rate and husbandry conditions. Kim et al [18] observed three MyHC-based fiber types in muscles of Korean native cattle, which was similar to the observation in this study. However, MyHC IIa had the highest expression followed by MyHC IIx in LT [18], which was different from the observation in this study. It seems that the discrepancies in results between studies are caused by the differences in species and breeds. The percentages of slow-MyHC I and fast-MyHC IIa and IIx in LT of TNC examined in this study (10.57%, 21.09%, and 68.34%, respectively) are comparable to the results of Waritthitham et al [19] who found that the LD muscles of Brahman×TNC had lower percentage of slow twitch fiber than fast twitch fiber (25.8% and 74.2%, respectively). Likewise, Kirchofer et al [20] reported that IF muscle containing β-red fibers greater than 40% were classified as red while LD muscles containing α-white fibers greater than 40% were classified as white. SS muscle was classified as intermediate due to the similar proportion of all fiber types. The relative differences in MyHC IIx and to a lesser extent MyHC I between KL and KI meat may reflect differences in growth rate and husbandry conditions between the TNC breeds. Similarly, differences in myofiber types were reported in different breeds of cattle [21].

The LT presented higher CP and ash contents, and lower moisture and fat contents than the SS and IF. These observations may be attributed to the differences in the expression of MyHC, reflecting the fiber type of the muscles. Similar to this study, Hwang et al [8] reported higher fat percentage in slow Psoas major than fast Semimembranosus. Conversely, a higher fat percentage was reported in fast LD [9]. The highest glycogen content at slaughter was found in the LT, which was concomitant with the lowest pH48. This finding agrees with those of previous studies, in which the slow twitch muscles with predominantly type I fibers had lower glycogen concentrations than those with type II or fast twitch muscles [22]. It appears that fast twitch muscles have high glycolytic capacity, which requires glycogen as a substrate. Glycogen concentration varies between the fiber types and this, combined with glycolytic capacity can influence the rate and extent of pH decline in muscles [23]. Onopiuk et al [24] reported a higher glycogen content in fast-twitch, glycolytic LD muscle (7.79 mg/g) than that in slow-twitch, oxidative Psoas major (7.10 mg/g) of beef cattle at 2 h postmortem. Moreover, the LD muscle had significantly lower pH_48_ (5.44) than Psoas major (5.53) [24]. The KL meat had lower CP and higher moisture content than did KI. Differences in growth rate and fiber types may be responsible for this observation. Differences in moisture and CP contents have been reported in different cattle breeds [21]. Fat content did not differ between breeds. Similarly, breed did not affect the fat percentage of beef [21]. Contrarily, fat percentage in native breed beef was lower than that of Brahman crossbred and Charolais crossbred beef [4].

Total and insoluble collagen contents were muscle dependent. The IF and SS had higher collagen content than the LT. Likewise, Torrescano et al [25] reported the amounts of collagen in different beef muscles as follows: IF>LD>PM while Rhee et al [26] reported a slightly different result: SS>IF>LD >PM. Because of the higher content of MyHC I, IF was the slowest fiber followed by SS while LT was the fastest muscle due to the higher content of MyHC IIx. Thus, it could be inferred that slow IF and SS muscles in beef cattle have higher collagen contents compared with the fast LT muscles. Insoluble and total collagen were positively correlated with MyHC I, and negatively correlated with MyHC IIx in the present study. The relationships between muscle fiber types and meat quality traits such as collagen [25] have been described previously.

The KL meat had higher lightness and lower redness and yellowness than the KI meat. The differences in MyHC IIx and MyHC I content and consequently the fiber type differences between breeds may explain this finding. Our findings are consistent with those of Sethakul et al [4], who reported that LD muscle of grass grazed native Thai cattle had lower lightness, yellowness, and redness values than the Charolais crossbred with heavy slaughter weight in an intensive system. Further, Brahman×Thai native beef had lower redness and yellowness than the Charolais×Thai native beef [14] Contrarily, Xie et al [21] observed no differences in the lightness and redness of beef in different cattle breeds. However, differences in meat yellowness were found in different cattle breeds [21]. The LT had higher L* value and lower a* and b* value when compared to other muscles. The lower b* in LT may reflect its lower intramuscular fat content. The differences in L* and a* may reflect the variation in MyHC isoforms between the muscles. According to Tortora [27], slow fibers have a high mitochondrial and capillary density and myoglobin content, although they are smaller in size and store less glycogen content than the fast-twitch fibers.

Of all the meat quality measurements, a significant interaction between breed and muscle type was detected for only pH_48_. The KI had a larger range in pH_48_ across muscles than the KL with the fast fiber type muscle LT having the lowest pH48 and the slow fiber type IF having the highest. This relationship was reflected in the correlation analysis where, pH_48_ was positively correlated with MyHC I and negatively correlated with MyHC IIx. Cooking loss did not differ among the muscles. Contrarily, Rhee et al [26] reported the following cooking losses for different beef muscles: SS (27.3%)>PM (23.6 %)>IF, LD (20.7%). There was significant effect of muscle on shear force measured at 48 h postmortem with LT having the highest value followed by SS then IF. This observation was unexpected given the lower total and insoluble collagen contents in LT compared with SS and IF. Nonetheless, the lower intramuscular fat may possibly account for the higher shear force in LT. Intramuscular fat in the perimysium could remodel the structure of the intramuscular connective tissue thus lowering the mechanical strength of intramuscular connective tissue and contributing to meat tenderness [28]. Our results contradict the findings of Rhee et al [26], who reported that the shear force values for beef muscles were as follows: SS (4.95 kg)>IF (3.99 kg)>LD (3.27 kg). However, this order does not always hold as Sullivan and Calkins [29] reported a slightly different result: SS (4.71 kg)>LD (4.20 kg)>IF (3.2 kg) in *Bos taurus* and their crossbred indicating that the relative order of shear force values can vary between muscles. The KI meat had higher shear force than the KL meat. This finding may be due to the higher cooking loss, lower moisture content, and higher calpastatin in KI compared with KL. An increase in cooking loss would probably reduce meat tenderness since a certain cross-sectional area of meat would comprise of less water and more structural components [5]. Calpastatin is capable of inhibiting the proteolytic activity of calpain, thereby reducing meat tenderness [13].

Post-mortem proteolysis plays a significant role in the development of meat quality, particularly tenderness. The relative percentage of the 30 kDa degradation product of troponin T at 48 h postmortem was significantly different among muscle types, with LT having the highest. This observation agrees with the findings of Christensen et al [30] who reported that postmortem glycolysis occurred faster in fast LD muscle than in slow vastus intermedius (VI) muscle, indicating that the proteolytic potential is greater in LD than in VI muscles. Our findings suggest that the differences in muscle fiber type are expected to have an impact on postmortem proteolysis and tenderization. Although there was a significant effect of muscle on shear force, there was no significant correlation between MyHC expression and the 30 kDa Troponin T degradation product. This infers that the mechanisms of meat tenderization are multifaceted. Despite the impact of breed on shear force, breed did not influence the 30 kDa degradation product of troponin T. Neither breed nor muscle influenced calpain 1 and calpain 2 activities. Nonetheless, KI muscle had higher calpastatin concentration than the KL muscle at 1 h postmortem. This observation indicates that there was a higher capacity to inhibit calpain activity in KI than in KL. There has been a consensus that calpain 1, calpain 2, and calpastatin are largely responsible for postmortem proteolysis in skeletal muscle and meat tenderization [31]. Although calpain 1 plays a major role in postmortem proteolysis [9,31], calpastatin is the specific endogenous inhibitor of calpain. Therefore, high concentrations of calpastatin at slaughter are known to be associated with tougher meat. A previous report has described a negative relationship between calpastatin level in muscle and meat tenderness [32]. A potential reason for higher calpastatin in KI muscles may reflect differences in the balance between protein synthesis and degradation between the species. A typically high muscle calpastatin expression has been shown to be associated with muscle hypertrophy and increased feed utilization efficiency; an extreme example of this is callipyge sheep, which has a very high calpastain expression [33]. Nonetheless, in this study, data on production parameters were not recorded but future work will hopefully assess these parameters.

Overall, the current results indicated that the differences in meat quality due to breed and muscle type of TNC are largely dependent on the distribution of MyHC isoforms. The impact of muscle type on meat quality of TNC was more pronounced than that of breed effect. The KL beef had lower shear force and cooking loss compared to the KI beef. Conversely, the KI beef presented higher redness than the KL beef. The LT muscle had higher shear force than the IF and SS. Thus, these results suggest that processing conditions such as grinding, slicing, and simmering that reduce toughness could be applied to KI meat and the LT muscle of TNC. As such, such meats could be utilized for meat products that require ground beef.

## CONCLUSION

The results of this study revealed that three MyHC isoforms namely MyHC I, MyHC IIa, and MyHC IIx were detected in IF, LT, and SS in TNC. Breed had no effect on the expression of MyHC I and MyHC IIa. The KL meat had higher MyHC IIx and fat content and lower calpastatin and shear force than the KI. The expression of MyHC I and MyHC IIx was different between muscles reflecting their fiber type with IF and LT, being slow and fast fiber types, respectively. The percentage of MyHC I and IIx were significantly correlated with selected muscle and meat quality traits. The current results indicated clear differences in meat quality between the KL and KI native cattle breeds in Thailand. These results would provide information that could be useful in the processing and utilization of meat from the KL and KI breeds of native Thai cattle.

## Figures and Tables

**Table 1:** Myosin heavy chain isoforms relative percentage expression in three muscle types (M) from two breeds (B) of Thai native cattle from central and northeastern parts of Thailand

Traits^1)^	Breed^2)^		Muscle^3)^			RMSE	p-value		
		
	KL	KI	IF	LT	SS		B	M	B×M
MyHC I	34.62	41.91	61.53^a^	10.57^c^	42.69^b^	11.69	0.051	<0.0001	0.340
MyHC IIa	24.72	23.11	25.54	21.09	25.11	8.04	0.519	0.285	0.564
MyHC IIx	40.66^a^	34.98^b^	12.93^c^	68.34^a^	32.20^b^	8.65	0.041	<0.0001	0.038

RMSE, root mean square error.

1) Arbitrary densitometry units/0.2 g of wet weight muscle.

2) KL, Kho-Lan (central origin); KI, Kho-Isaan (northeastern origin).

3) IF, *Infraspinatus*; LT, *Longissimus thoracis*; SS, *Supraspinatus*.

a–c LS means having different superscripts along the same row within each main effect are significantly different (p<0.05).

**Table 2:** Calpain system and Troponin T degradation product in three muscle types (M) from two breeds (B) of Thai native cattle from central and northeastern parts of Thailand

Traits	Breed^1)^		Muscle^2)^			RMSE	p-value		
		
	KL	KI	IF	LT	SS		B	M	B×M
Calpain activity^3)^									
Calpain 1	5.23	5.02	4.97	5.16	5.26	0.64	0.285	0.490	0.707
Calpain 2	4.58	4.56	4.61	4.53	4.57	0.56	0.922	0.936	0.689
Calpastatin^4)^	0.20^b^	0.25^a^	0.23	0.22	0.22	0.06	0.006	0.964	0.081
Troponin T degradation product^5)^	48.17	47.67	47.70^b^	50.21^a^	45.84^b^	2.24	0.594	0.004	0.886

RMSE, root mean square error.

1) KL, Kho-Lan (central origin); KI, Kho-Isaan (northeastern origin).

2) IF, *Infraspinatus*; LT, *Longissimus thoracis*; SS, *Supraspinatus*.

3) Arbitrary densitometry units (AU)/0.2 g of wet weight muscle.

4) Arbitrary absorbance values of calpastatin/100 μg protein.

5) Total intensity of intact and degradation products of Troponin T within each sample is taken as 100%. Values indicated are relative percentage of the signal intensity of degradation product.

a,b LS means having different superscripts along the same row within each main effect are significantly different (p<0.05).

**Table 3:** Meat characteristics of three muscle types (M) from two breeds (B) of Thai native cattle from central and northeastern parts of Thailand

Trait	Breed^1)^		Muscle^2)^			RMSE	p-value		
		
	KL	KI	IF	LT	SS		B	M	B×M
Crude protein (%)	19.35^b^	20.29^a^	19.16^b^	20.62^a^	19.69^b^	0.56	<0.0001	<0.0001	0.674
Ether extract (%)	1.03	0.93	1.22^a^	0.68^b^	1.03^a^	0.30	0.316	0.0002	0.672
Moisture (%)	78.31^a^	77.07^b^	78.02^a^	77.06^b^	77.99^a^	0.57	<0.0001	<0.0001	0.254
Ash (%)	1.12	1.13	1.10^b^	1.17^a^	1.11^b^	0.038	0.202	<0.0001	0.346
Glycogen (mg/g wet weight)	25.58	31.89	28.27^b^	35.06^a^	22.88^b^	11.48	0.066	0.037	0.465
Collagen (mg/g wet weight)^3)^									
SC	0.55	0.49	0.53	0.42	0.61	0.19	0.309	0.052	0.599
IC	5.41	5.81	5.82^a^	4.35^b^	6.66^a^	1.60	0.429	0.002	0.860
TC	5.96	6.30	6.35^a^	4.78^b^	7.27^a^	1.73	0.534	0.002	0.924
Color									
L*	38.20^a^	32.72^b^	33.19^b^	36.64^a^	36.55^a^	2.94	<0.0001	0.005	0.814
a*	12.69^b^	14.73^a^	16.40^a^	10.62^c^	14.11^b^	1.52	0.0001	<0.0001	0.094
b*	13.57	14.32	14.83^a^	12.64^b^	14.38^a^	1.22	0.053	<0.0001	0.183
pH_48_	5.81	5.78	6.03^a^	5.43^b^	5.93^ab^	0.16	0.614	<0.0001	0.023
Cooking loss (%)	41.17^b^	45.56^a^	41.68	45.09	43.32	4.76	0.013	0.208	0.343
Shear force (kg)	7.03^b^	9.11^a^	6.22^c^	10.08^a^	7.92^b^	1.50	<0.0001	<0.0001	0.338

RMSE, root mean square error

1) KL, Kho-Lan (central origin); KI, Kho-Isaan (northeastern origin).

2) IF, *Infraspinatus*; LT, *Longissimus thoracis*; SS, *Supraspinatus*.

3) SC, soluble collagen; IC, insoluble collagen; TC, total collagen.

a,b LS means having different superscripts along the same row within each main effect are significantly different (p<0.05).

**Table 4:** Relationship between relative percentage of myosin heavy chain isoforms and meat characteristics of Thai native cattle

Trait	MyHC I	MyHC IIa	MyHC IIx
Crude protein (%)	−0.46^**^	−0.18	0.50^**^
Ether extract (%)	0.53^**^	0.20	−0.58^**^
Moisture (%)	0.31^*^	−0.04	−0.29
Ash (%)	−0.52^**^	−0.10	0.54^**^
Glycogen (mg/g wet weight)	−0.23	−0.21	0.29
Soluble collagen (mg/g wet weight)	0.20	0.28	−0.28
Insoluble collagen (mg/g wet weight)	0.44^**^	0.13	−0.47^**^
Total collagen (mg/g wet weight)	0.43^**^	0.15	−0.46^**^
L*	−0.33^*^	−0.16	0.37^*^
a*	0.79^**^	0.23	−0.84^**^
b*	0.66^**^	0.07	−0.66^**^
pH_48_	0.73^**^	0.30	−0.80^**^
Shear force (kg)	−0.46^**^	−0.30	0.54^**^
Cooking loss (%)	−0.15	−0.06	0.16
Calpain 1 activity^1)^	−0.03	−0.08	0.05
Calpain 2 activity^1)^	0.20	−0.23	−0.12
Calpastatin^2)^	0.09	−0.30^*^	0.01
Troponin T degradation product^3)^	−0.38	−0.34	0.45^*^

* p<0.05,

** p<0.01.

1) Arbitrary densitometry units (AU)/0.2 g of wet weight muscle.

2) Arbitrary absorbance values of calpastatin/100 μg protein.

3) Total intensity of intact and degradation products of Troponin T within each sample is taken as 100%. Values indicated are relative percentage of the signal intensity of degradation product.
